# Effect of Infant Prematurity on Auditory Brainstem Response at Preschool Age

**Published:** 2013

**Authors:** Sara Hasani, Zahra Jafari

**Affiliations:** 1 *Department of Audiology, Faculty of Rehabilitation, Tehran University of Medical Sciences, Tehran, Iran.*; 2 *Rehabilitation Research Center, Faculty of Rehabilitation, Tehran University of Medical Sciences, Tehran, Iran.*

**Keywords:** Auditory brainstem responses, Child, Click stimuli, Latency, Premature, Infant

## Abstract

**Introduction:**

Preterm birth is a risk factor for a number of conditions that requires comprehensive examination. Our study was designed to investigate the impact of preterm birth on the processing of auditory stimuli and brain structures at the brainstem level at a preschool age.

**Materials and Methods:**

An auditory brainstem response (ABR) test was performed with low rates of stimuli in 60 children aged 4 to 6 years. Thirty subjects had been born following a very preterm labor or late-preterm labor and 30 control subjects had been born following a full-term labor.

**Results:**

Significant differences in the ABR test result were observed in terms of the inter-peak intervals of the I–III and III–V waves, and the absolute latency of the III wave (P<0.019). No significant differences were observed in the amplitude of the I or V waves. The choice of test ear examined had no effect on the results.

**Conclusion:**

Our results indicate an effect of preterm birth on ABRs and synchronization of auditory stimuli at preschool age, and suggest the need for further follow-up over the coming years.

## Introduction

The neurological development of newborns involves both the central and peripheral nervous system. The central nervous system continues to grow up to an age of 3 years, and gradually develops over the following years to reach maturity. The peripheral nervous system consists of peripheral nerves, such as hearing and balance nerves, with a period of development that is longer than for other nerves (1). 

A study in Iran performed in two groups with normal and abnormal development determined that birth before 37 weeks is associated with defects in development (2). Although rates of delivery before 34 weeks’ gestation have remained relatively stable since 1990, rates of delivery during the late-preterm period (typically defined as 34 –36 weeks’ gestation) have increased by 20% between 1990 and 2006 in the United States (US) (3). 

According to the US Academy of Obstetrics and Gynecology, preterm birth (birth before 37 weeks of age) is called the embryonic period. Moderately preterm neonates are born at 32 to 36 weeks and very preterm infants are born at less than 32 weeks (4). Over 70–75% of preterm infants and 9% of all births in the US are moderately preterm infants (5). The embryonic brain (including structures and neural pathways) exhibits rapid growth between 34 to 36 weeks’ gestation. Thirty-five percent of the embryonic brain and 47% of the volume of the cortex is formed during the sixth week of pregnancy. Myelinization and an increase in the gray-matter volume of brain occurs at a rate of 1.4% between 34 to 41 weeks. Since the preterm infant is born before 37 weeks’ gestation and this brain development occurs outside the uterus, the formation of the neural network will be different. Several physiological, psycholo- gical and environmental risk factors such as disease states, medical treatments, care environments and caregiver characteristics and their interactions can impede healthy transition from utero, compound existing biological vulnerabilities, and therefore, adversely influence the progression of growth and development (5). The weight of the brain of a preterm infant is almost 65% that of a full-term baby, and also has fewer surface grooves. This lack of maturity in the infant brain can cause considerable damage (6). Preterm infants are at risk of difficulties including intellectual delay, speech articulation problems, attention deficits hyperactive disorders (ADHD), and specific learning disorders affecting reading, writing, and mathematics, cerebral palsy, long-term disease, and anti-social behavior (1,2,7–9). Preterm birth in some cases has led to reductions to 5–15% of perfect movement and approximately 25–50% in the educational process, affecting family life (9). In this regard, Luu and colleagues (2011) and Woodward and colleagues (2009) noted that individuals with a history of preterm birth are at greater risk of problems with executive skills, cerebral palsy, cognitive delay, language delay, and emotional and behavioral adjustment problems and multiple disorders compared with controls (8,9). Neponnyaschy and colleagues (2011) reported cognitive disorders, lower scores in mathematics, reading and writing in children with a history of late-preterm birth at 2 and 4 years of age compared with a normal group (10). Karlsson and colleagues (2010) reported a lower performance in the neuro-cognitive abilities and learning of children with a history of very low birth weight when compared with the control group (11).

Preterm children have an organic complic- ation in the nervous systems; thus it is important to examine the auditory central nervous system to identify and reduce complications of hidden hearing loss. The standard method to examine the auditory system at the brainstem level is auditory brainstem response (ABR). Zhi and colleagues observed longer inter-peak intervals in the ABR test after increasing the rate of stimuli in children with a history of preterm birth compared with the control group, suggesting a mild delay in neural transmission in the brainstem (12). Ribeiro and colleagues showed longer latency in ABR testing with tone-burst stimuli in premature infants compared with controls (13), while Pasman and colleagues reached the same findings in children of 5 years of age with a history of preterm birth (14). According to studies, reviewing of the brainstem in infants and children is possible and safe (13,14). Jiang and colleagues reported a delay or impairment in function of the brainstem of a very preterm infant (15).

Preterm birth in the US is the largest public health problem. 

According to many magnetic resonance imaging (MRI) studies in children and adults with a history of preterm birth, the size of the cortical gray and white matters and overall brain size in individuals with a history of preterm birth are smaller than control ones (1,16–19). Immaturity of the brain has a major role in neurological disability in older age. MRI experiments show that the brain weight of newborn infants born at 35 weeks is 65% that of a baby born at full term. This lack of maturity may increase the vulnerability of the brain later in life (20–22). Many studies have confirmed that preschool children with cognitive and language delays, behavioral and emotional problems, as well as substantial adaptation and multiple disorders have a lower intelligence quotient (IQ) (1,3,8,10,11,23–27). 

Most studies are performed at birth, but because the central nervous system continues to grow to 3 years and still develops gradually over the following years to reach maturity, so the study of brain structures and neural networks at the preschool age through auditory-evoked responses are more accurate and give us useful information. Recently, the US has suggested the need for studies of the neurological and behavioral outcomes of children with a history of preterm birth (28). Hence, because of the paucity of data in this area and to address this public health concern, this study was performed to assess the effects of a history of preterm birth on auditory stimulus processing at the brainstem level in children aged 4–6 years. The preschool age was chosen because it is at this age that children enter public and classical education; thus children at risk of hearing loss should be screened for potentially subtle hearing disorders in order to study the impact on subsequent educational and social skills.

## Materials and Methods

Our study was performed in 30 children, including 16 boys and 14 girls, with a history of preterm birth with an average age of 57.13 months (standard deviation [SD], 6.93 months; range, 48–72 months). All children were recruited from the audiology clinic of faculty of rehabilitation between September and December 2011. Thirty normal controls with an average age of 63.06 months (SD, 6.96 months; range, 51–73 months) were also recruited. 

To study the effect of preterm birth on maturation of ABR waves, children with a preterm history were divided to two groups: very preterm (VP; less than 32 weeks) and late preterm (LP; 32 to 36 weeks). The VP group consisted of 14 children (five boys and nine girls) and the LP group consisted of 16 children (11 boys and five girls).

To eliminate the confounding effect of neurological abnormalities on the result of preterm birth, only children who had normal neurological development were included in our study. Children with a history of preterm birth were selected by a review of medical documents from 4 to 6 years previously at the Hedayat Hospital in Tehran, while normal matched children were selected from the same age from a kindergarten. Inclusion criteria included normal peripheral hearing, parents' satisfaction, no history of use ototoxic drugs, recurrent ear infections, high fever, head trauma, surgical, and psychological problems (based on parents' speech). To ensure the absence of any abnormalities or external ear and middle ear diseases and children with normal peripheral hearing, all cases underwent an otoscopy examination, tympanometry, and acoustic reflex test (Intracoustic AD229h). 

In addition, pure-tone and speech audiometry were performed in a double-walled, sound-treated audiometric booth, using a clinical audiometer (Intracoustic AC40) and a supra-aural headphone (Telephonics TDH-39P). This study was approved by the ethics committee of Tehran University of Medical Sciences.

ABR was recorded using an Intracoustic Eclipse instrument via four surface electrodes; non-inverting electrode on the vertex, two inverting electrodes on mastoid in both sides and a reference electrode on the forehead using a 15-ms time window, alternate stimulus polarity, two rates of 21.1 and 51.1 c/s and 2000 sweeps in the supine position with eyes closed. Before connecting the electrodes, the skin was cleaned thoroughly to ensure good contact between the skin and the electrode surfaces. The absolute electrical impedance was less than 5 k.ohm, with no more than 2 k.ohm difference between each of the two electrodes. 

In addition, masking was not used in our recordings. On the other hand, to confirm the ABR result in each ear, good morphology and appropriate test-retest reliability of the waves including maximum 2-ms changes in absolute latencies and maximum 20% changes in amplitudes of the five principle ABR waves were considered.

The click stimulus had a duration of 100 µs, with an intensity of 80 dB nHL presented by insert receivers, while a 30-dB white noise was used to mask the contra-lateral ear. Average responses were 2000 sweep. 

A Colmogrov-Smirnov test was used to define the normal distribution of data. An independent t-test was used to compare the mean value of the results. For statistical analysis, Spss.17 software was used at a 0.05 significance level.

## Results

The choice of test ear examined had no effect on the results (P>0.082); therefore both ears were considered together (N=60 years in each group).

**Fig1:**
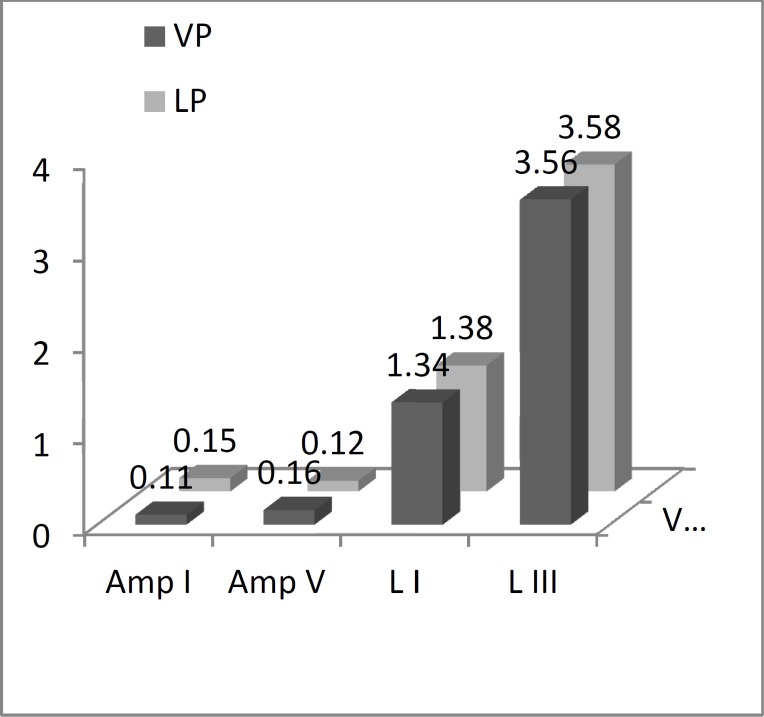
Comparison of mean amplitudes of auditory brainstem response I and V waves and absolute latencies of I and III peaks between late preterm and very preterm groups

There was no significant difference between the LP and VP groups except in the mean amplitude of wave I at a high stimulus rate (P=0.02) (Fig. 1). 

In the subsequent analysis, moderate preterm and VP children with a history of preterm birth were considered to be one group for the purposes of comparison with normal term children.

**Fig2:**
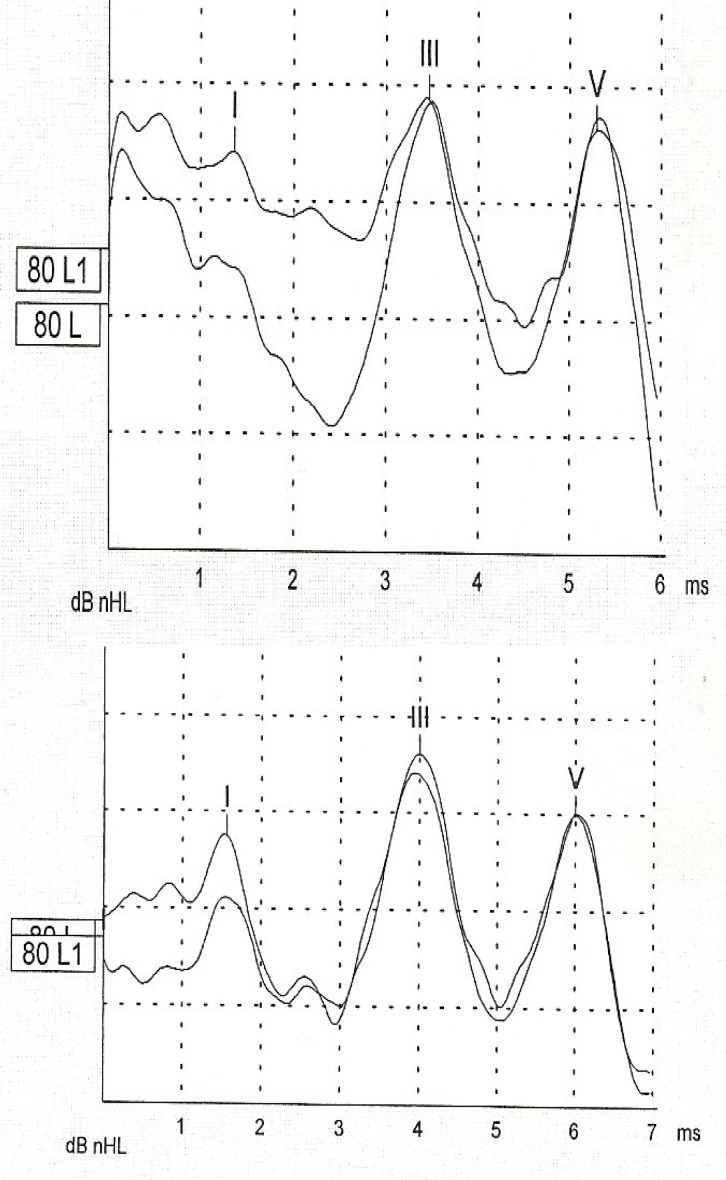
Comparison of auditory brainstem response test results between normal and preterm children

Significant differences were observed in inter-peak intervals of I–III, III–V and absolute latency of the III wave (Table 1) (Fig. 2).

**Table 1:** Comparison of absolute latencies and inter-peak intervals of ABR waves between normal and preterm children

ABR waves (ms)	Premature children		Normal children		P-value
	Mean	SD	Mean	SD	
Inter-peak latency of I–III	2.20	0.11	2.07	0.20	0.00
Inter-peak latency of III–V	1.84	0.13	1.92	0.12	0.002
Inter-peak latency of I–V	4.00	0.32	3.99	0.23	0.793
Absolute latency of I wave	1.36	0.10	1.41	0.15	0.055
Absolute latency of III wave	3.57	0.17	3.49	0.19	0.019
Absolute latency of V wave	5.36	0.36	5.41	0.23	0.348

## Discussion

In this study, the click ABR test was recorded with low stimulus rates in normal children and children with a preterm history. No notable difference was observed between the left and right ears in each group. A study by Amorim and colleagues, among 86 infants who were divided into premature infants and normal infants and then divided into four groups based on chronological age (0 to 29 days, 30 days to 5 months, and 29 days and older than 6 months) also reported similar results (16). It seems that the maturation process occurs similarly in both sides.

In comparing data between two groups of children with late and very early preterm birth, there were no notable differences in the other parameters. A study of Pasman and colleagues in children with a history of preterm birth showed longer latencies of I and III waves than in children with a history of very preterm birth, and they observed a significantly longer latency of the I wave at an age of 5 years in very preterm children (14). Given the low number of studies in this field, studies with a larger sample size could allow more precise conclusions in this regard.

In our study, ABR test parameters measured in children with a history of preterm birth were compared with those measured in normal children. Often mean latencies of ABR in preterm children were longer than in the normal group. These differences were noticeable especially in the inter-peak intervals of the I–III, III–V waves and absolute latencies of the III wave. Longer latencies of premature neonates can be attributed to their early maturity. Similar results have also previously been reported in earlier studies. Based on previous studies, latencies decreased as a result of the child growing, and latency is therefore a suitable indicator to detect the evolution process of the central and peripheral auditory system (13,31–33). Pasman and colleagues investigated the effects of very preterm and late-preterm birth on the ABR at 40 and 52 weeks of age and at 5 years of age (14). Absolute latencies of the II _C_, III, V, and V waves of the ABR test in neonates at 52 weeks of age were remarkably longer than for the controls. Also, the late-preterm group showed longer inter-peak latencies of the I–III and IV waves at 52 weeks of age compared with very preterm children and the normal group. In this study, middle ear infection with a delay in myelinization of the central auditory pathway was noted as a cause of these differences (14). The increase in the inter-peak interval of I–V is in agreement with the findings of previous studies by Kuttenr, Eggermont, and Pasman (14,30,31). However, in studies by Kaga and colleagues and Amorim and colleagues no noticeable differences were observed in the absolute latencies of the I, III, and V waves and in the intervals between the ABR waves (16,17). 

Amorim offered the low sample numbers in the groups of preterm neonates as a cause of this result (16). Recent studies further confirm the results of our study (12–15,29). Amorim reported that the absolute latency of I wave is close to the value in adults; suggesting that maturation of the auditory nerve occurs in the first month of life and there is a gradual reduction in the latencies of the III and V waves as a result of child growing, reflecting the maturity of the axons and synapses in the brain stem (16).

In explaining the findings of studies on preterm birth and their effects on ABR, the mechanisms that are involved in the maturation of the neurophysiologic auditory system need to be discussed. These mechanisms include the growth of the external and middle ears and the cochlear, myelinization of the axons, dendrite growth, and increase in synaptic activity (37–41). The cochlea is completed at approximately the 20^th^ week of life and the peripheral system of hearing pathways reaches its growth limit in the first weeks of life. Synapses are formed in the late months of pregnancy and dendrites are completed after birth. Myelinization starts at 6–12 months of life and continues for several years later. Myelinization of the vestibular ways and auditory brainstem occur before birth with high speed, while myelinization of other systems occurs later and at lower speeds (42–48). Longer absolute and inter-peak intervals of the ABR waves in preterm children compared with normal children supports the hypothesis of delay and deficit in the myelinization of infants and children with a history of preterm birth.

As expected, the amplitude of the I and V waves did not differ between the two groups of children. This indicator has not been studied in most previous studies (13-15,29). Zhi and colleagues also reported similar results in the amplitude of the I wave (12). Amplitude has a low volatility but large variability, especially at young ages. Individual variability is large and therefore fewer studies considered it. In general, there are several reasons for the variability of this parameter. The electric fields of the ABR are weak and the electrical activity in surface of the skin is very small because amplitude originates in the brainstem structures located deep within the skull. The amplitude or recorded electric field depends on the distance and direction of the nerve and recording electrodes. Thus, individual differences in neuroanatomy and anatomy such as the size of the head (50), the thickness of the skull (51), and skin impedance (30) affect the amplitude (52). The amplitude and absolute and inter-peak intervals of the I and V waves in children with a history of preterm birth was similar to those from normal. Children with a history of preterm birth showed considerably longer inter-peak latencies of the I–III, III–V waves and in absolute latency of the III wave at a rate of 21.1 c/s compared with the normal group. Zhi and colleagues reported remarkable differences in the inter-peak latency of the III–V waves between the two groups (12). However, no notable differences were reported in the amplitude of the I wave or the inter-peal latency of the I-V waves as a result of differences attributed to delays in transmission of auditory brainstem neurons in many parts of the central pathways in children with a history of preterm birth (12). Results of this study provide normal values for ABR variables ​​in healthy children at the age of 4 to 6 years and provide justification for further investigation of other risk factors for hearing on the electrophysiological and behavioral responses. These results could inform health professionals about the impact of counseling and therapy on reducing the effect of risk factors. Fundamental-application studies such as this can enhance knowledge and information about the impact of risk factors on hearing loss and growth on auditory nerve pathways. With regard to conflicting results in the ABR test between children with a history of LP and VP births, performing studies on a larger sample size as well as studies of larger brain areas using late tests such as event-dependent auditory responses (LLR, P300, MMN) would provide more comprehensive and more insightful results.

## Conclusions

According to this study, differences were seen in ABR results between preterm and term children, and the latency of waves in the preterm group was greater than in the control group. Therefore, it seems that there is a defect in many parts of the central auditory brainstem that should be followed up in order to detect patients that require a hearing aid to decrease educational and social problems. 
